# Surgical management of inverted papilloma; a single-center analysis of 247 patients with long follow-up

**DOI:** 10.1186/s40463-017-0246-7

**Published:** 2017-12-20

**Authors:** Oisín Bugter, Dominiek André Monserez, Floris Vincent Willem Joseph van Zijl, Robert Jan Baatenburg de Jong, Jose Angelito Hardillo

**Affiliations:** Department of Otorhinolaryngology and Head and Neck Surgery, Erasmus MC, ‘s-Gravendijkwal 230, 3015 CE, Rotterdam, The Netherlands

**Keywords:** Inverted papilloma, Endoscopic surgery, Recurrence, Retrospective study, Follow-up

## Abstract

**Background:**

Our aim was to review our management of inverted papilloma (IP), perform a recurrence analysis, and review the literature.

**Methods:**

A retrospective analysis of 247 patients treated for an IP. Patients were grouped according to surgical approach, tumor presentation (primary, residual and recurrence) and Krouse-stage.

**Results:**

Recurrence was observed in 20.3%, 28.6% and 35.1% (*p* = 0.017) of the patients who underwent endoscopic, external and combined surgery, respectively. Recurrences occurred more often in residual than primary IP (36.9% vs. 22.3%, *p* = 0.021). Primary endoscopic surgery had a recurrence rate of 12.5%, which was comparable to the recent literature (11.2%, 161/1433).

**Conclusions:**

The relatively high number of recurrences in this cohort is explained by the long follow-up and previous (incomplete) surgery in 61.5% of the cases. The inferior outcome of residual IP underscores the importance of having a low threshold for preoperative biopsy in unilateral and atypical sinonasal disease.

## Background

Inverted papilloma (IP) is a benign tumor of the sinonasal cavities. It was first described by Ward more than 150 years ago as a follicular tumor involving the nasal bones [1]. It took until 1938 for Ringertz to recognize the characteristic endophytic growth pattern and introduce the term ‘inverting papilloma’ [2]. Since then many researchers have contributed to the extensive knowledge we now have on IP [3].

The incidence of IP ranges from 0.2 to 0.6 people diagnosed per 100,000 per year. The most frequently reported complaints are similar to those of sinonasal polyps: unilateral nasal obstruction and rhinorrhea. IP is most commonly diagnosed in the fifth and sixth decade of life and has a predilection for males with a ratio of 3:1 [4]. The exact etiology of IP is unclear, but there are indications that HPV infection plays a role in the progression of inverted papilloma and confers an increased risk for recurrence and malignant transformation [5, 6]. The frequency of IP in unsuspected sinonasal polyps is reported to be between 0.31 and 0.93% [7, 8].

The standard treatment has always been surgery aimed at complete removal of the tumor. Its association with malignancies in 5–15% is one of the distinct characteristics of IP that endorses surgical treatment [9]. Other important features include its aggressive growth, that causes adjacent bone to locally erode, and its tendency to recur. Over the last decades endoscopic surgery has become the gold standard for the treatment of IP with many studies showing significantly better treatment outcome in patients treated endoscopically [9].

The aim of this study was to analyze the developments in our management of IP over the last three decades, in particular with regard to the recurrence rates and outcomes of treatment. All surgical approaches were reviewed, with an emphasis on endoscopic surgery. Whether the presentation of the tumor (primary, residual or recurrent) before surgery in our clinic would affect the recurrence rate also had our interest. Finally, a literature review of IP cohort studies was performed to compare with our surgical outcomes and recurrence rates.

## Methods

The Ethics Committee of the Erasmus Medical Center, a tertiary referral medical center, stated that ethical permission was not required for this retrospective study.

We included 323 IP patients, of which 247 were included in the recurrence analysis, between January 1983 and December 2012. All patients were obtained from PALGA, a Dutch nationwide network and registry of histo- and cytopathology. All cases received surgical treatment, had a preoperative histopathological proven diagnosis of IP, and complete clinical information. Clinical data was assessed using the electronic medical records (CSC-iSOFT, Virginia, USA) and paper charts.

The IP was classified as a primary when no prior surgery was performed and diagnosis was confirmed by a simple biopsy. Patients with previous surgery were divided into two groups: residual and recurrent IP. Residual IP were defined as those who underwent functional endoscopic surgery for reasons other than IP, which was deemed as inadequate tumor surgery. Inverted papilloma diagnosis is this group was unexpected at postoperative histopathological examination. Recurrent IP were defined as those who underwent adequate tumor surgery in a different, non-academic hospital with the preoperative suspicion or diagnosis of IP. These patients were referred to us for revision surgery after diagnosis of a recurrence.

All patients underwent surgery by experienced head and neck surgeons. Surgical treatment was divided into three groups: endoscopic surgery (pure endonasal endoscopic approach), external surgery (e.g., Denker, lateral rhinotomy, osteoplastic flap) and combined approaches (endoscopic surgery combined with Caldwell-Luc). The extent of surgery and approach were chosen based on the perceived origin and extent of the IP. In the endoscopic group, we mainly performed a medial maxillectomy, including (at least the posterior two thirds of) the inferior turbinate and an ethmoidectomy. We opted for a radical ethmoidectomy, including middle turbinate and opening of the frontal recess, for tumors limited to the ethmoid and/or frontal recess. For the external approach, we mainly performed a classic Denker (medial maxillectomy via opening of the anterior wall of the maxillary sinus, complete removal of maxillary sinus mucosa, radical ethmoidectomy, and sphenoidectomy). The combined approach was applied to maxillary tumors originating lateral to the infraorbital nerve. A medial maxillectomy was performed in all cases with the exception of tumors limited to only the frontal sinus or sphenoid. Complications after surgery were classified according to the Clavien-Dindo Classification of Surgical Complications (Table 1) [10]. All patients were followed up by endoscopy every three months for the first two years. After this period, patients were followed up biannually for up to five years. MRI and/or biopsies were performed when a recurrence was suspected.

**Table 1:** Summary of Clavien-Dindo Classification of Surgical Complications

Grade I	Any deviation from normale post-operative care without the need for treatment or interventions
Grade II	Requiring pharmacological treatment or blood transfusion
Grade III	Requiring surgical, endoscopic or radiological intervention
Grade IV	Life-treatening complication requiring IC/ICU management
Grade V	Death of a patient

A literature search was performed of EMBASE (2005, jan – 2016, dec) using the key term ‘inverted papilloma’. All IP case series with a minimal follow-up of 12 months, reporting on recurrence rates per operative approach, and published in the English language were included.

### Statistical analysis

When quantitative variables were normally distributed, the results were expressed as mean values and standard deviation (SD), otherwise median and inter quartile range (IQR). Differences between two groups were analyzed using the t-test or the Mann-Whitney U test. Qualitative data are reported as counts and frequencies, and differences between groups were analyzed using the chi-squared test or Fisher’s exact test. Multivariate logistic regression was used to investigate the association of Krouse staging, surgical approach and presentation of tumor and the risk of recurrence [11]. Cox regression analysis was performed to evaluate the association between variables of the univariate analysis and recurrence free survival time. Statistical analysis was performed using SPSS version 21 (IBM Co., Armonk, NY, USA) and cut off point for significance was *p* < 0.05.

## Results

The mean age at time of diagnosis was 55.8 years (SD 13.7). The study population consisted of 245 males and 78 females (3.1, 1). Nasal obstruction was the most frequent complaint (257 patients, 79.6%). Other complaints were rhinorrhea (73, 22.6%), headache (38, 11.8%), epiphora (21, 6.5%) and feeling of pressure in the face or forehead (17, 5.3%). Thirty patients (9.3%) had no complaints. Preoperative radiological imaging was performed with CT-scan in 201 cases (62.2%) and with MRI in 15 cases (4.6%). Both CT and MRI were used in 62 patients (19.2%). Forty-five patients (13.9%) underwent surgery without preoperative radiological imaging. The left and right side of the sinonasal cavity were equally involved (50.2% vs. 48.3%). Five patients (1.5%) had bilateral involvement.

In 121 of the cases (37.5%) the inverted papilloma was primary, in 115 cases (35.6%) residual, and in 87 cases (26.9%) recurrent. The tumors were classified as Krouse T1 to T4 in respectively 38 (11.8%), 148 (45.8%), 108 (33.4%) and 29 cases (9.0%). Twenty-eight IP were classified as T4 because of an associated malignancy and one because of extrasinonasal extension. Endoscopic surgery was performed in 107 patients (33.1%), a combined approach in 22 cases (6.8%) and an external procedure in 194 patients (60.1%).

### Complications

The overall percentage of perioperative complications was 14.6% (47 cases). Most complications were Clavien-Dindo grade I (24, 51.1%), such as sensibility disorders, perioperatively managed cerebrospinal fluid leakage and excessive crustae. Ten patients (21.3%), including four wound infections, had grade II complications. Most Clavien-Dindo grade III complications (10, 21.3%) were complaints of epiphora after a classical Denker procedure that required a dacrocystorhinostomy. Two patients (4.3%, grade IV) developed meningitis post-operatively and had to be admitted to the intensive care unit. One patient died post-operatively as a result of severe comorbidity (2.1%, grade V).

### Malignancies

Thirty-one patients (9.6%) had an associated malignancy. The malignancy occurred synchronously in 28 cases (90.3%). They consisted of 25 squamous cell carcinomas (89.3%), two verrucous carcinomas (7.2%), and one undifferentiated carcinoma (3.6%). Three other cases squamous cell carcinomas developed metachronously (9.7%).

Four patients (12.9%) had a carcinoma in situ. Nine patients had tumor with T stage I (29.0%) three with II (9.7%), three with III (9.7%) and 12 with IV (38.7%). Three patients (9.7%) had a regional lymph node metastasis and three patients (9.7%) a distant metastasis. Most patients (80.6%) with an associated malignancy were treated with an external approach. For patients with an associated malignancy, the 5-year overall survival was 80.6% and the 5-year disease specific survival 87.1%. Eight patients with an a associated malignancy (25.8%) developed a recurrent IP after initial treatment. The 5-year recurrence free survival rate was 77.4%. During follow-up 20 patients (64.5%) remained alive with no evidence of disease. One (3.2%) was alive with disease, eight (25.8%) died of disease, and two (6.5%) died of other cause.

### Recurrence analysis

We included a sub selection of 247 patients cohort in our recurrence analysis. Sixty-one patient were excluded because their follow up was <2 years. Another fifteen patients with suspected residual IP were excluded because postoperative histopathologic examination did not confirm the residual IP. The median follow-up was 66.2 months (IQR 41.1–112.9). Overall recurrences occurred in 74 patients (30.0%)(Table 2).

**Table 2:** Multivariate analysis of recurrence rates according to surgical approach and presentation of the tumor

	Endoscopic approach	Combined approach	External approach	Total
	*No. of recurrences /* *total no. of patients (%)*	*No. of recurrences /* *total no. of patients (%)*	*No. of recurrences /* *total no. of patients (%)*	*No. of recurrences /* *total no. of patients (%)*
Primary IP	3 / 24 (12.5)	1 / 3 (33.3)	18 / 68 (26.5)	22 / 95 (23.2)
Residual IP	8 / 32 (25.0)	2 / 6 (33.3)	21 / 46 (45.7)*	31 / 84 (36.9)†
Recurrent IP	5 / 23 (21.7)	1 / 5 (20.0)	15 / 40 (37.5)	21 / 68 (30.9)
Total	16 / 79 (20.3)	4 / 14 (28.6)	54 / 154 (35.1)‡	74 / 247 (30.0)

*p = 0.021 compared to primary IP treated with external approach. † *p* = 0.028 compared to total primary IP. ‡ *p* = 0.017 compared to total endoscopic approach

#### Type of surgery

Seventy-nine (32.0%) cases in our recurrence analysis were treated with pure endonasal endoscopic surgery. Recurrences occurred in sixteen endoscopic cases (20.3%). Primary IP treated endoscopically recurred in three cases (12.5%), residual IP in eight cases (25.0%) and recurrent IP in 5 cases (21.7%). Four of fourteen patients (28.6%) treated with a combined approach developed a recurrence. The majority of the cases had an external procedure (154, 62.3%). They developed recurrences in 54 cases (35.1%). This is significantly higher than the endoscopic group (*p* = 0.017, OR 95 CI 2.3 [1.1–4.4]) (Table 2). Primary tumors treated externally recurred in eighteen cases (26.5%). For residual (45.7%, *p* = 0.028, OR 95% CI 2.4 [1.1–5.6]) and recurrent (37.5%) tumors, this percentage was higher. The distribution of Krouse stage was not significantly different in these groups (Table 3).

**Table 3:** Distribution of Krouse stage per surgical approach

Krouse stage	Endoscopic approach	Combined approach	External approach	Total
	*no. of patients (%)*	*no. of patients (%)*	*no. of patients (%)*	*no. of patients (%)*
T1	15 (19.0)	0 (0.0)	13 (8.4)	28 (11.3)
T2	39 (49.4)	2 (14.3)	75 (48.7)	116 (47.0)
T3	22 (27.8)	11 (78.6)	49 (31.8)	82 (33.2)
T4	3 (3.8)	1 (7.1)	17 (11.0)	21 (8.5)
Total	79 (100.0)	14 (100.0)	154 (100.0)	247 (100.0)

#### Presentation of tumor

The risk of recurrence was significantly higher in the residual IP group than primary IP (31 [36.9%] vs 22 [23.2%], *p* = 0.021 OR 95% CI 2.2 [1.1–4.3]). Recurrence occurred in 21 cases (30.9%) in the recurrent IP group.

#### Krouse stage

Krouse stage did not influence the overall recurrence rate significantly. Patients with Krouse stage T1-T4 IP developed recurrences in respectively 7 (22.6%), 36 (30.5%), 25 (28.9%), and 6 (42.9%) cases.

#### Time to recurrence

The median time to recurrence was 20.5 months (IQR 11.8–37.5). The surgical approach and the presentation of the tumor had no significant influence on the time to recurrence. Table 4 gives an overview of when the recurrences occur during follow-up. Only forty-one (55.4%) of all recurrences were diagnosed in the first two years of follow-up. More than 80% (58) of all recurrences were diagnosed within four years.

**Table 4:** Time to recurrence

Follow-up	Endoscopic approach (*n* = 79)	Combined approach (*n* = 14)	External approach (*n* = 154)
	*No. of recurrences /* *total no. of recurrences (%)*	*No. of recurrences /* *total no. of recurrences (%)*	*No. of recurrences /* *total no. of recurrences (%)*
< 1 year	5 / 16 (31.3)	0 / 4 (0.0)	16 / 54 (29.6)
< 2 year	11 / 16 (68.8)	3 / 4 (75.0)	27 / 54 (50.0)
< 3 year	13 / 16 (81.3)	4 / 4 (100.0)	38 / 54 (70.4)
< 4 year	13 / 16 (81.3)		44 / 54 (81.5)
< 5 year	13 / 16 (81.3)		45 / 54 (83.3)
6–10 year	16 / 16 (100.0)		51 / 54 (94.4)
> 10 year			54 / 54 (100.0)

#### Number of recurrences

Thirty-seven (50%) of all 74 cases with a recurrence after initial surgery in our hospital developed a second recurrence after revision surgery. This occurred more often in the group of patients that underwent external surgery than the endoscopic group (5/16 [31.3%] vs. 32/54 [59.3%], *p* = 0.049). Eighteen patients (24.3%) developed a third recurrence, while six patients (8.1%) developed even more than three recurrences during follow-up.

### Literature review

A total of 64 articles IP cohort-studies between 2005 and 2016 were found, of which 23 articles with a total of 1989 patients met the inclusion criteria (Table 5) [12–34]. Their mean follow-up was 54.5 months. Of the 1433 patients treated endoscopically 161 (11.2%) developed a recurrence. Recurrence rates reported varied between 0.0% and 30.0%. Seven articles reported a total of 32 (12.3%) recurrences in 260 patients treated with a combined approach. Fifty-four (18.2%) recurrences occurred in a total of 296 external procedures. Fourty-one articles did not meet the inclusion criteria.

**Table 5:** Review of inverted papilloma cohort studies per surgical approach with a minimal follow-up of 12 months (2005–2016)

Authors	Year published	Endoscopic approach		Combined approach		External approach		Follow-up (months)		
		*No. of recurrences / %)*		*No. of recurrences /* *total no. of patients (%)*		*No. of recurrences /* *total no. of patients (%)*		*Mean*	*Low*	*High*
Poetker et al. [12]	2005	0 / 8	(0.0)	1 / 4	(25.0)			29.8	12	80
Kamel et al. [13]	2005	3 / 52	(5.8)					65	24	160
Peng et al. [14]	2006					2 / 98	(2.0)	61	24	168
Minovi et al. [15]	2006	6 / 61	(9.8)	3 / 20	(15.0)	0 / 6	(0.0)	74	12	175
Mirza et al. [16]	2007	4 / 32	(12.5)			4 / 16	(25.0)	60	12	240
Harvinder et al. [17]	2008	0 / 5	(0.0)					23	12	36
Jurado-ramos et al. [18]	2008	4 / 34	(11.8)			8 / 18	(44.4)	54.3	12	60
Durucu et al. [19]	2009	2 / 23	(8.7)	1 / 14	(7.1)	3 / 19	(15.8)	35	16	42
Sham et al. [20]	2009	12 / 40	(30.0)			6 / 16	(37.5)	84	24	216
Rutherford et al. [21]	2010	0 / 2	(0.0)					19	15	24
Giotakis et al. [22]	2010	5 / 39	(12.8)			11 / 25	(44.0)	91	36	146
Gras-Cabrerizo et al. [23]	2010	10 / 57	(17.5)			7 / 22	(31.8)		12	
Pagella et al. [24]	2011	1 / 20	(5.0)					50	24	87
Dragonetti et al. [25]	2011	5 / 84	(6.0)					39	13	97
Lombardi et al. [26]	2011	12 / 198	(6.1)	0 / 14	(0.0)			53.8	24	192
Kim et al. [27]	2012	64 / 372	(17.2)	17 / 144	(11.8)	10 / 62	(16.1)	41	12	
Pagella et al. [28]	2014	1 / 73	(1.4)					58	18	138
Sciaretta et al. [29]	2014	7 / 103	(6.8)					57.7	24	167
Gu et al. [30]	2014	1 / 7	(14.3)			3 / 14	(21.4)	65	36	144
Erbek [31]	2015	0 / 8	(0.0)					30.8	12	60
Hong [32]	2015	0 / 6	(0.0)	3 / 25	(12.0)			55.6	36	93
Adriaensen [33]	2016	15 / 121	(12.4)	7 / 39	(17.9)				12	139
Healy [34]	2016	9 / 88	(10.2)					97.2	36	
Total		161 / 1433	(11.2)	32 / 260	(12.3)	54 / 296	(18.2)	54.5		

## Discussion

We have, to our knowledge, presented the largest ever single-center cohort of inverted papilloma patients. Our reported recurrence rates were higher for residual and recurrent IP than primary IP. We also confirmed superiority of endoscopic surgery over combined and external approaches. Finally, we’ve shown that many recurrences occur more than two years after initial treatment.

Thirty percent of all the patients in our recurrence analysis developed a recurrence (Table 2). This is relatively high compared to recent literature (Table 4). This could be explained by our heterogeneous cohort with a high number (62.3%) of external surgery. Also our rate is based on results covering a large time period with one third of the patients treated before the ‘endoscopic era’. These patients account for more than half of all recurrences. Based on our observation, most of the recurrences after an external approach, Denker in particular, occur in the frontal recess and ethmoidal roof. Tumor remnants at these areas can easily be missed or left out during such procedures due to lack of good visualization and magnification. Nowadays, we make it a point to inspect these areas using an endoscope and drill out as much bone as possible. Several literature reviews on IP recurrences have been published between 1981 and 2009 [9, 14, 35–37]. Their average recurrence rates after endoscopic surgery vary between 12.5% and 19.6%. This is higher than the 11.3% in our review of more recent studies, suggesting a global decrease in recurrence rates in endoscopic IP surgery.

Our recurrence in the endoscopic group (20.3%) was also higher than the average 11.2% (161/1433) in our review of the recent literature (Table 5). However, primary tumors treated by endoscopically had a similar recurrence rate of 12.5%. Therefore, we believe that the relatively high recurrence rate is due to our high number of residual and recurrent IP and to our long follow (median > six years). After splitting the complete endoscopic group in a 2003–2007 (the first years of endoscopic IP surgery in our clinic) and a 2008–2012 cohort, we also noted a non-significant learning curve is noticed with 31.8% and 15.4% recurrences (*p* = 0.12) respectively.

A wide range of recurrence rates (0.0–45.0%) have been documented after revision surgery (Table 6) [20, 26–29, 33, 38–40]. However, it is generally accepted that it has a worse outcome than primary surgery, which is support that functional endoscopic sinus surgery should not be used as a diagnostic tool for unilateral sinonasal disease (a pre-operative biopsy should). The worse outcome of revision IP surgery has been explained due to the increased difficulty to identify the attachment site of the IP because of distorted anatomy and the absence of landmarks [33, 41, 42]. Post-operative inflammation of the remaining sinonasal mucosa could also attribute to this due to an increased difficulty to distinguish normal mucosa from tumor. This is most evident in patients with a residual IP due to the relative short time between primary and revision surgery. It could even be that incomplete surgery has an effect on the inflammatory pathway of the etiology and progression of IP, possibly explaining multi-centered recurrent IP.

**Table 6:** Recurrence rates according to presentation of the tumor in eight cohort studies

Authors	Year published	Primary cases		Revision cases	
		*No. of recurrences /* *total no. of patients (%)*		*No. of recurrences /* *total no. of patients (%)*	
Tufano et al. [40]	1999	1 / 17	(5,9)	4 / 16	(23,2)
Han et al. [39]	2001	0 / 14	(0,0)	3 / 17	(17,6)
Lee et al. [38]	2004			3 / 17	(17,6)
Sham et al. [20]	2009	9 / 36	(25,0)	9 / 20	(45,0)
Lombardi et al. [26]	2011	8 / 156	(5,1)	4 / 56	(7,1)
Kim et al. [27]	2012	70 / 456	(15,4)	21 / 122	(17,2)
Pagella et al. [28]	2014	1 / 64	(1,6)	0 / 7	(0,0)
Sciarretta et al. [29]	2014	0 / 57	(0,0)	7 / 53	(13,2)
Gu [30]	2014			4 / 21	(19.0)
Adriaensen et al. [33]	2015	2 / 49	(4,1)	13 / 72	(18,1)

Many articles claim that the majority of recurrences after IP-surgery occur in the first two years [20, 43]. Sham et al. published a detailed data of time to recurrences of a group of 56 patients, which supported this statement [20]. This was however not the case in our larger cohort: only 55.4% of all recurrences occurred in the first two years (Table 4). This percentage was slightly higher (68.8%) in patient treated endoscopically. A follow-up of three years after endoscopic surgery and four years for all cases was needed to diagnose >80% of the recurrences. A multi-center study has also shown a significant difference in recurrence rate between patients with a follow-up longer and shorter than three years (26.1 vs. 8.5%) [27]. Therefore, our department supports suggestions by Suh and Adriaensen to perform a long or even life-time clinical follow-up [33, 43].

## Conclusion

The recurrence rate of residual tumors is significantly higher than primary tumors. Therefore clinicians should have a low threshold for suspecting IP and when uncertain first take a biopsy. Furthermore, given the mean time to recurrence and the rate of late recurrences a long follow-up is required in both the clinic and research.

## Abbrevation

IP: Inverted papilloma
